# A case report of multinodular hepatic steatosis mimicking pseudotumors of the liver

**DOI:** 10.4102/sajr.v26i1.2410

**Published:** 2022-06-27

**Authors:** Pavel Burko, Nitin Juggath, Ruslan Iliasov, Mariya Fedorova, Natalia Nazarova

**Affiliations:** 1 Department of Diagnostic Radiology, National Medical Research Center of Rehabilitation and Balneology, Moscow, Russian Federation; 2 Department of Diagnostic Radiology, Clinic for Diagnostics and Management on Izmaylova, Penza, Russian Federation; 3 Department of Radiation Medicine, Faculty of Health Sciences, University of Cape Town, Cape Town, South Africa; 4 Department of Surgery, Clinic for Diagnostics and Management on Izmaylova, Penza, Russian Federation; 5 Department of Morphology, Faculty of General Medicine, Penza State University, Institute of Medicine, Penza, Russian Federation; 6 Department of Radiology, Penza Institute for Further Training of Physicians - Branch Campus of the Russian Medical Academy of Continuous Professional Education, Penza, Russian Federation; 7 Department of Diagnostic Radiology, Clinical Hospital №6 named after G.A. Zakharyin, Penza, Russian Federation

**Keywords:** liver, hepatic steatosis, pseudotumours, magnetic resonance imaging, computed tomography, fatty liver disease

## Abstract

Fatty liver disease (FLD) is a common, benign pathology often found incidentally. We present a clinical case in which metastatic liver disease was suspected on initial imaging studies. Following further investigations, a diagnosis of ‘non-alcoholic fatty liver disease (NAFLD), multinodular type’ was postulated. Subsequent histology confirmed the presence of liver steatosis. Multinodular type hepatic steatosis is a rare, but clinically important pathology to identify and differentiate from other multifocal lesions of the liver parenchyma.

## Introduction

Fatty liver disease (FLD) is an excessive accumulation of fatty acids in hepatocytes in the form of triacylglycerol, usually predominantly around the central veins (hypoxic area) in the absence of inflammation or liver damage. It is one of the most common chronic liver diseases. According to a large meta-analysis performed in 2016, which included more than 8 million people from 22 countries, fatty liver infiltration occurs in 25.24% of the world’s inhabitants.^1^ According to the data of the largest Russian study DIREG 2, NAFLD occurs amongst outpatients of medical preventive institutions in 37.30%.^2^ There is also evidence that this is the most common disease of the internal organs in the Russian Federation.^3^

Simple steatosis (excessive accumulation of fat in the form of triglycerides in the liver), observed in NAFLD, is often benign. However, progression of the disease to non-alcoholic steatohepatitis increases the risk of developing fibrosis, cirrhosis, liver failure, hepatocellular carcinoma and, accordingly, increases the risk of death.^1^

The gold standard for diagnosing FLD is a percutaneous puncture biopsy followed by histological analysis.^4,5^ However, this is an invasive technique, at risk of various complications. Accordingly, the systematisation of non-invasive diagnostic algorithms is one of the most pressing problems of modern hepatology and internal medicine.

Although it should be noted that none of the techniques, such as ultrasound (the appearance of increased echogenicity of the liver due to fatty infiltration, resulting in increased hepatic/renal and hepatic/vascular gradient; can have other aetiologies), CT (hypodensity of the liver parenchyma is not completely specific for fatty tissue, especially in cases of the ‘patchy’ type and with indolent lipid accumulation) and MRI (decrease of signal on out-of-phase gradient echo images is characteristic for FLD; however, during analysis of the diffuse form, global signal decrease may be a pitfall in interpretation) in isolation cannot provide an absolute certainty in the diagnosis of FLD in all clinical cases.

The diagnosis of FLD based on images is usually straightforward, but sometimes fat accumulation can show unusual structural patterns that can mimic neoplastic, inflammatory or vascular pathology. In these cases, the macrostructure of the liver visualised by imaging techniques can lead to an incorrect diagnosis, unnecessary diagnostic tests and invasive procedures.

This report describes a case of multinodular (‘patchy’) type of steatosis, confirmed by radiological and pathological analysis. The imaging appearance of the disease mimicked metastatic liver disease, which led to clinical dissonance in determining the strategy of patient management.

## Case report

A 34-year-old male patient sought consultation in the Clinic for Diagnostics and Management on Izmaylova (Penza, Russia) in accordance with a previously completed workup in order to determine the next management steps.

Physical examination revealed no abnormalities. Recorded vital signs were: blood pressure 115/85 mm Hg, heart rate 75 beats/min, temperature 36.7 °C and respiratory rate 20 breaths/min.  His saturation was 98%. Results of abdominal examinations were normal. There was no history of alcohol consumption, diabetes mellitus, drug therapy or viral hepatitis C.

**FIGURE 1:**
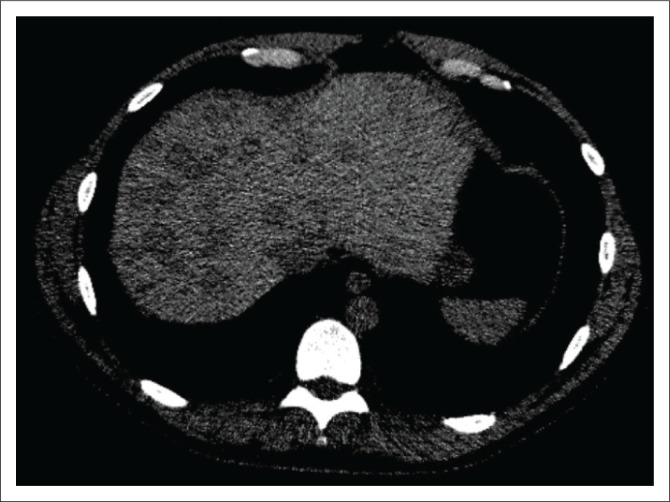
Non-contrast CT imaging of the chest revealed a multinodular (‘patchy’) pattern of the lesions in the hepatic parenchyma.

Chest CT was performed independently at another clinic. A CD archive of the CT scan without contrast enhancement was provided for a second-opinion. CT analysis demonstrated no pathological changes in the lung parenchyma. At the lower margin of the field of view, a heterogeneous liver parenchyma was noted with numerous variable-sized hypodense nodular foci of round or oval shape in an unequal distribution. The maximum diameter was 2.5 cm. Density reading of these liver lesions was about +30 HU (absolute low attenuation), with background liver parenchyma density measuring +50 HU. Furthermore, there was no associated mass effect. Vascular structures (hepatic veins and portal branches) traversed the nodules without disruption (Figure 1). The obvious etiological identity of the lesions was not determined. In accordance with the data from the CT scans, elastography for assessment of the degree of liver fibrosis, MRI with contrast for further characterisation of the nodules and evaluation of the laboratory profile was planned.

Laboratory analysis data included alpha-fetoprotein, carcino-embryonic antigen, CA19-9, CA 72-4, neuron-specific enolase and CYFRA 21-1, which were not abnormal. Other laboratory findings were as follows: alanine transaminase (ALT): 43.8 IU/L, aspartate transaminase (AST): 36.7 IU/L, AST/ALT ratio (De Ritis coefficient): 0.8, total bilirubin: 8.9 µmol/L. Platelets were 267 × 10^3^/µL. AST to platelet ratio index (APRI) was 0.35 (negative predictive value for cirrhosis).

The real-time shear wave elastography (SWE) data was obtained. The area of interest was located at a depth of 2 cm – 4 cm from the liver capsule. The stage of liver fibrosis was assessed using the METAVIR F0-F4 scale in accordance with the recommendations of the European Federation for Ultrasound in Medicine and Biology. The distribution of colour shades in all segments was relatively uniform. The average elasticity was 7.67 kPa (1.6 m/s), which corresponded with stage F2 (moderate fibrosis).

**FIGURE 2:**
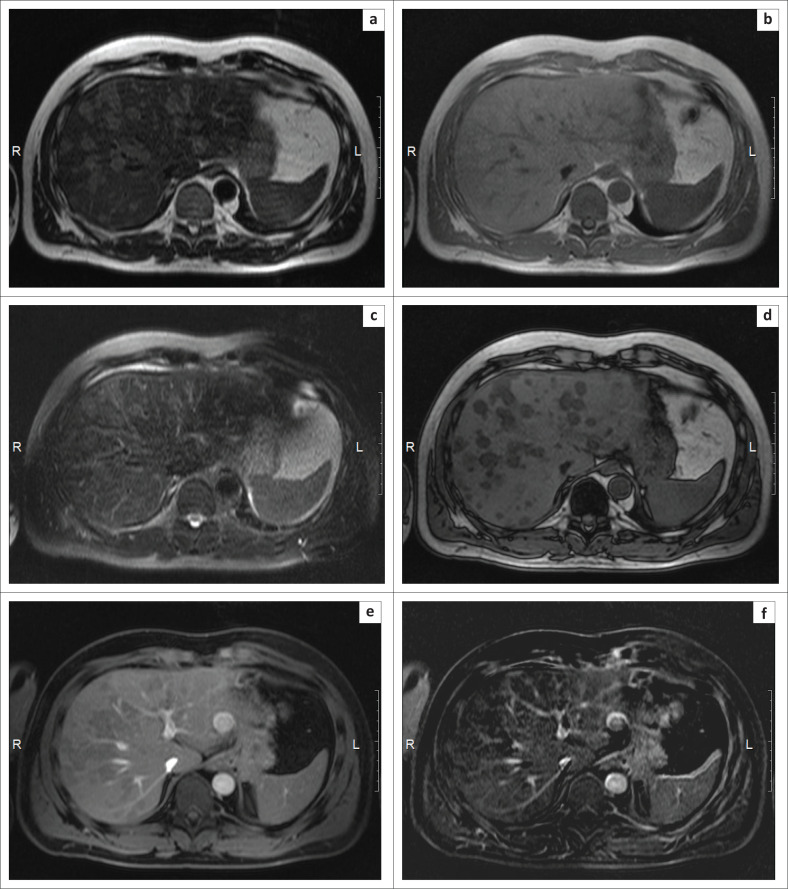
Contrast-enhanced abdominal MRI demonstrated a multinodular (‘patchy’) appearance in the hepatic parenchyma: (a) T2-weighted image; (b) in-phase gradient echo image; (c) T2-FatSat-weighted image; (d) out-of-phase gradient echo image; (e) T1-weighted 3D gradient echo LAVA-flex sequence (post-gadolinium) and (f) subtracted image.

Contrast-enhanced abdominal MRI was performed. Similar findings were appreciated as described earlier on CT. The multiple foci in the hepatic parenchyma were slightly hyperintense on T2- and T1-weighted images. There was some suppression on T2-FatSat. On dual gradient echo in-phase and opposed phase (chemical shift selective imaging sequences), a characteristic inversion of the MR signal was detectable due to the intracellular lipid content in the structure of the foci (MR signal drop from pathological (microscopic) fat on the T1-weighted out-of-phase). A quantitative assessment of the fat in the liver was carried out on the basis of the two-point Dixon method with flexible echo times, followed by a grading of liver steatosis depending on the percentage of fat according to the schema outlined by Kleiner et al.^6^ A reading of 34% was obtained, which corresponds to grade 2 (moderate fatty hepatosis). The lesions demonstrated no contrast enhancement or diffusion restriction and there was no mass effect from the foci (Figure 2).

In accordance with the data obtained, a preliminary diagnosis of non-alcoholic fatty liver disease was made which led to the decision to execute a percutaneous liver biopsy followed by histological analysis.

**FIGURE 3:**
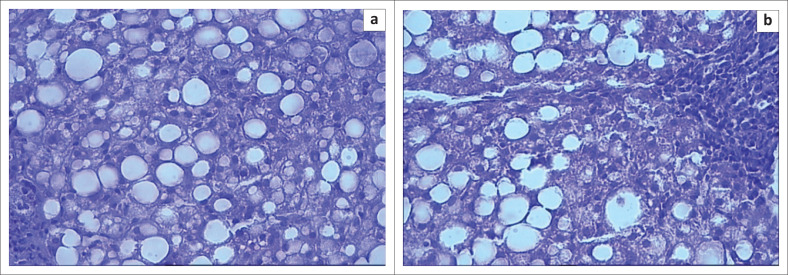
Sectioned slides. Macrovesicular globules of fat, inflammation in the area of the periportal tract, focal fibrosis (hematoxylin and eosin, × 100).

Pathological analysis in accordance with the scoring system recommended by Brunt et al. (2001)^7^ was performed. Primarily, the analysis was focused on determining the degree of steatosis, the activity of inflammation and the stage of liver fibrosis. Histologically, the specimen stained with hematoxylin and eosin, (× 100) showed pronounced fatty degeneration. More than 66% of hepatocytes were affected, which corresponded to third-degree macrovesicular (large droplet) steatosis (Figure 3) – a very pronounced steatosis, but at the same time, the inflammatory changes are moderate. Focal round-cell infiltration in the area of the periportal tracts was also identified. These changes corresponded to the second degree of steatohepatitis. Focal fibrosis was differentiated in the third acinus zone, defined as the first stage of fibrosis.

After histopathological analysis, a clinical diagnosis of non-alcoholic fatty liver disease: steatohepatitis (second degree) and fibrosis of liver (first stage) was made.

## Discussion

Fatty liver disease is caused by abnormal lipid accumulation in liver cells. It has a relatively benign course, however, as soon as inflammation occurs, the risk of developing fibrosis and cirrhosis increases.^8^ Of note, there is a widespread prevalence of this pathological condition in the population and a rather frequent identification when performing imaging. Of greatest interest is ‘patchy’ or multinodular hepatic steatosis, which is the rarest in comparison with focal and diffuse forms, and quite often causes difficulties in differential diagnosis as it mimics metastatic liver disease or pseudotumours.^9,10^

The pathogenesis of liver steatosis is based on the accumulation of an excessive amount of hepatocellular lipids, represented by triglycerides and other cholesterol derivatives in hepatocytes, due to an imbalance between the synthesis and utilisation of these organic molecules.^11^ The accumulation of fatty droplets in the liver may be the result of an excessive supply of free fatty acids in the liver or their own increased synthesis by the liver from acetylcoenzyme A, especially in the case of a surplus of the acetylated form of coenzyme A.^12^

The main clinical feature of FLD is asymptomaticity. Most often, the disease is detected randomly on the basis of laboratory or instrumental data. In some, clinical symptoms can be recognised as manifestations of metabolic syndrome, such as visceral obesity, signs of impaired glucose metabolism, dyslipidemia and arterial hypertension. The physical examination has no pathognomonic features.

The key points in the diagnosis of FLD are attributed to imaging techniques. Sensitivity and specificity for CT without contrast in the identification of liver steatosis are 33% and 100%, respectively, for CT with contrast, 50% and 83%, respectively, and for MRI, 88% and 63%, respectively.^13^

Ultimately, a histological analysis remains the gold standard for the diagnosis of FLD.^4,5^ This is relevant in view of the possibility of obtaining detailed information about the histopathology of the liver, despite the disadvantages of the method, such as invasiveness, high cost and limited study area (especially with respect to diffuse lesions). The diagnosis depends on the qualifications of the pathologist, the risks of complications, and the possibility of erroneous interpretation in the case of an unsatisfactory sample.

We collectively decided to execute histological verification, rather than dynamic observation, based on clinical features. From our point of view, it was more ideal for the patient discussed in this report. In addition, this made it possible to confirm the NAFLD with a high degree of reliability, to make a differential diagnosis between steatosis and steatohepatitis, to evaluate the stage of fibrosis and, based on the results of histological analysis, to make a forecast on the further course of the disease. As a result, our preliminary diagnosis coincided with the pathologist’s opinion. This suggests that FLD can be diagnosed at the preinvasive stage of management with a high degree of probability by the rational organisation of laboratory and instrumental techniques, even if an abnormal pattern of FLD is detected.

## Conclusion

Fatty liver disease is a widespread pathological condition, which is often detected at imaging. The most prevalent imaging pattern is diffuse. Less common types of hepatic steatosis are focal, perivascular, subcapsular and multinodular. They can mimic neoplastic, inflammatory or vascular conditions, which can lead to confusion, unnecessary diagnostic tests and invasive procedures. Therefore, multinodular (‘patchy’) hepatic steatosis is a pathology that we must include in the differential diagnosis of nodular liver lesions. The gold standard for diagnosis is percutaneous liver biopsy with histological analysis. However, it should be noted that liver fat deposition can be diagnosed non-invasively by ultrasound, CT or MRI if the established criteria are used. In many cases, this allows the diagnosis of FLD without the need for invasive procedures.
